# A novel messenger RNA signature as a prognostic biomarker for predicting relapse in pancreatic ductal adenocarcinoma

**DOI:** 10.18632/oncotarget.22861

**Published:** 2017-12-02

**Authors:** Guodong Shi, Jingjing Zhang, Zipeng Lu, Dongfang Liu, Yang Wu, Pengfei Wu, Jie Yin, Hao Yuan, Qicong Zhu, Lei Chen, Yue Fu, Yunpeng Peng, Yan Wang, Kuirong Jiang, Yi Miao

**Affiliations:** ^1^ Pancreas Center, Department of General Surgery, The First Affiliated Hospital of Nanjing Medical University, Nanjing 210029, China; ^2^ Pancreas Institute of Nanjing Medical University, Nanjing 210029, China; ^3^ Endoscopy Center, The First Affiliated Hospital of Nanjing Medical University, Nanjing 210029, China

**Keywords:** predictive signature, biomarker, bioinformatics, pancreatic ductal adenocarcinoma, relapse-free survival

## Abstract

Pancreatic ductal adenocarcinoma (PDAC) death rate and recurrence rate have remained obstinately high. Current methods can not satisfy the need of predicting cancer relapse accurately. Utilizing expression profiles of 10 GEO datasets (*N* = 774), we identified 154 differentially expressed genes (DEGs) between PDAC and normal pancreas tissue or paracancerous tissue. Next we built a 16-mRNA-based signature by means of the LASSO COX regression model. We also validated the prognostic value of the signature. Patients were classified into high-risk and low-risk group according to the signature risk score; 1 year RFS was 45% (95% CI: 31.6%–63.9%) for high-risk group in contrast to 92.5% (95% CI: 86.3%–99.1%) for low-risk group. Moreover, it could predict RFS well in cases with the receipt of different treatment modalities. The 16-mRNA-based signature was an independent and powerful prognostic biomarker for RFS for PDAC patients (HR = 7.74, 95% CI: 3.25–18.45, *p* < 0.0001).

## INTRODUCTION

PDAC accounts for approximately 90% of pancreatic cancer (PC), which is the third leading cause of cancer mortality followed by lung cancer and colorectal cancer [1]. 5 year overall survival (OS) for PC has increased slightly from 5% to 8% and more than 52% cases are initially diagnosed at a distant stage for which 5 year OS is only 3% [1].The main reasons include the shortcomings of effective therapies and the lack of specific biomarkers or clinical symptoms in diagnosis.

Many therapeutic fields have emerged for decades, such as surgery, postoperative adjuvant chemoradiotherapy, targeted molecular therapy (TMT), neo-adjuvant chemotherapy, immunotherapy, and therapeutic exosomes or microvesicles. Surgical resection is still the only potentially curative therapy. However, less than 20% have a surgical opportunity and over one-half of cases experience a postoperative relapse [2]. Cancer relapse directly results in shorter survival time after operation. Adjuvant chemotherapy with gemcitabine has been a standard care for resected PC which can delay recurrence [3].

Tumor tissue and serum samples contain massive potential diagnostic and prognostic biomarkers. Proteins and nucleic acid molecules including mRNAs, microRNAs, long non-coding RNAs and stable circular RNAs from tissue samples, and free molecules consisting of above mentioned molecules, tumor-derived exosomes or microvesicles, circulating tumor cells and circulating tumor DNA from serum samples have been explored as specific biomarkers for different tumors.

Gene Expression Omnibus (GEO) and The Cancer Genome Atlas (TCGA) are two main public databases that provide massive array-based and sequence-based data for global researchers to download [4, 5]. Meanwhile, novel bioinformatic methods make it quicker and more capable to deal with large amounts of data. To date, previous methods can’t satisfied the need of predicting cancer relapse accurately. Although studies have identified some biomarkers for PDAC by integrating GEO with TCGA, most of them focused on overall survival instead of relapse-free survival. In our study, we carefully reviewed all datasets about PDAC and the eligible datasets were more comprehensive. We used R software to analyze gene expression levels and identify significant genes which expressed differentially between PDACs and normal pancreas tissue (TvsN) or paracancerous tissue (TvsP) [6]. Gene Ontology (GO) enrichment analysis and Kyoto Encyclopedia of Genes and Genomes (KEGG) pathway analysis were performed for finding out key genes and pathways. Furthermore, we utilized Least absolute shrinkage and selection operator (LASSO) regression model and built a 16-mRNA-based signature for predicting the present of relapse. The model was validated in 10-foldcross-validation [7]. The prognostic value of the signature was tested by the performance of multiple analyses.

## RESULTS

### Identification of differentially expressed genes

**Figure 1:**
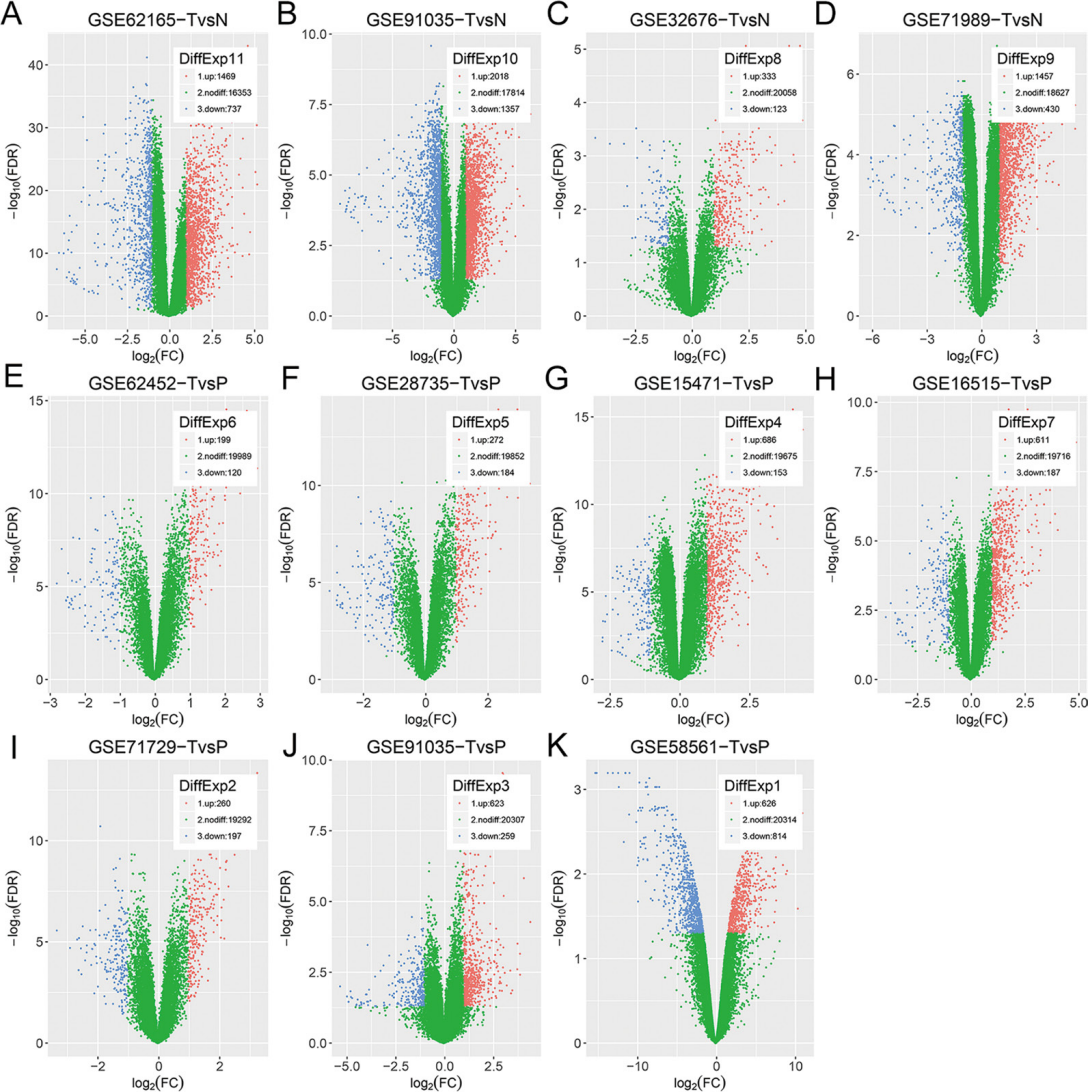
Vocano plots of DEGs in selected datasets (**A–K**) GSE91035 consists of gene expression profiles of not only TvsN but also TvsP. X-axis: log[2](FC); Y-axis: -log[10](FDR) for each gene. Genes with FDR < 0.05 and absolute FC > 2 are considered as DEGs in each series. Blue: down-regulated genes; Green: non-differential genes; Red: up-regulated genes.

**Figure 2:**
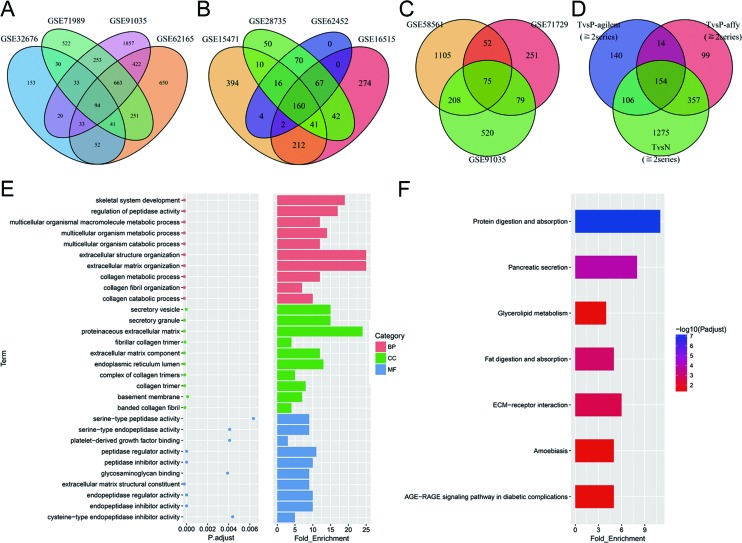
DEGs between PDAC and normal pancreas tissue or paracancerous tissue (**A**–**D**) Overlap analysis in 11 different datasets. Every ellipse corresponds to a dataset. The numbers of DEGs in each overlapped area are marked in relevant position. DEGs within 2 dataset or more were regarded as credible in each venn diagram. (**E**) Top 10 terms in each of three categories of GO enrichment analysis. Red: biological processes (BP); Green: cellular component (CC); Blue: molecular functions (MF). (**F**) KEGG pathway analysis of DEGs. Seven signal pathways are significantly enriched.

All eligible datasets were described in Table 1. Vocano plots of DEGs in these datasets were displayed in Figure 1. In addition, overlapping analysis of these DEGs was conducted. Four gene expression profiles were utilized to recognize DEGs of TvsN (Figure 2A). There were other 7 gene expression profiles used to identify DEGs of TvsP, including 3 gene expression profiles on agilent microarrays and 4 gene expression profiles on affymetrix microarrays (Figure 2B–2C). Due to the larger number of datasets, DEGs within 2 series or more were regarded as credible in each venn diagram. As shown in Figure 2D, there were 154 overlapping genes differently expressed among TvsN (≥2series), TvsP-affymetrix (≥2series) and TvsP-agilent (≥2series) finally. Of these, 103 genes were up-regulated mRNAs and 51 genes were down-regulated.

**Table 1:** Datasets enrolled in the study

GEO ID or data source	T	P	N	Platform
GSE62165 (Janky *et al*, 2016)	118	-	13	Affymetrix U219
GSE91035 (Schmittgen *et al*, 2016)	27	15	8	Agilent LincRNA G3
GSE32676 (Donahue *et al*, 2011)	25	-	7	Affymetrix U133 Plus 2
GSE71989 (Schmittgen *et al*, 2015)	13	-	8	Affymetrix U133 Plus 2
GSE62452 (Yang *et al*, 2016)	69	61	-	Affymetrix Gene 1.0 ST
GSE28735 (Zhang *et al*, 2012)	45	45	-	Affymetrix Gene 1.0 ST
GSE15471 (Badea *et al*, 2009)	36	36	-	Affymetrix U133 Plus 2
GSE16515 (Pei *et al*, 2009)	36	16	-	Affymetrix U133 Plus 2
GSE71729 (Moffitt *et al*, 2015)	145	46	-	Agilent G4112F
GSE58561 (Wennerström *et al*, 2014)	3	2	-	Agilent G3
TCGA-PAAD (The Cancer Genome Atlas Research Network, 2014)	138	-	-	Illumina HiSeq V2

N: normal pancreas tissue samples; P: paracancerous tissue samples; PAAD: patients with pancreatic adenocarcinoma; T: tumor samples.

### Results of GO enrichment analysis and KEGG pathway analysis

GO analysis and KEGG analysis were conducted for exploring the biological roles of these 154 DEGs. We listed top 10 terms in each of three categories (Figure 2E). Metabolic process was the main term of biological processes (BP), while terms associated with collagen and extracellular matrix (ECM) were enriched in cellular component (CC). Seven terms of molecular functions (MF) had relation with peptidase activity. As exhibited in Figure 2F, seven pathways were significantly enriched, of which protein digestion and absorption pathway was the most. The result of GO analysis integrated with KEGG analysis supported the point that ECM remodeling and pancreatic tumor-secreted microvesicles or exosomes promoted pre-metastatic niches formation and PDAC progression [8].

### Clinical characteristics of PDAC patients enrolled in the study

One hundred and thirty-eight PDAC patients with RFS status were enrolled in our study. Clinical characteristics of these patients were summarized in Supplementary Table 1. The median age of these patients was 65 years and median follow-up was 15.8 months. Thirty-nine of 138 patients experienced cancer relapse, of which 37 cases relapsed within 2 years. Following the AJCC 8th edition staging system, we had reassessed AJCC8 T stage, N stage and pathologic stage according to recorded maximum tumor dimension and the number of PLN (Supplementary Tables 2–4). Seventy-two T stage tumors, 46 N stage tumors and 62 pathologic stage tumors in the old system were reclassified into other stages in the 8th version system.

### Construction and validation of the prognostic signature

For these 154 candidate mRNAs, we used Nearest Neighbor Estimation (NNE) method to plot time-dependent ROC curves. The predict time was equal to 24 months. We generated the optimal cutoff value and other indices of each mRNA, such as the area under the curve (AUC), Protective or Risky factor (PorR), sensitivity and specificity. According to the cutoff value, 138 patients were classified into high or low expression status of this mRNA. Next we conducted COX univariable analysis between each mRNA and RFS. These *p* values were recorded. AUC ≥ 0.55 was a restrictive condition for filtering some mRNAs that hardly had a prognostic value. Ultimately, 65 mRNAs with *p* < 0.25 and AUC ≥ 0.55 were utilized to construct LASSO COX regression model (Supplementary Table 5). Of these, 53 genes were up-regulated mRNAs and 12 genes were down-regulated.

**Figure 3:**
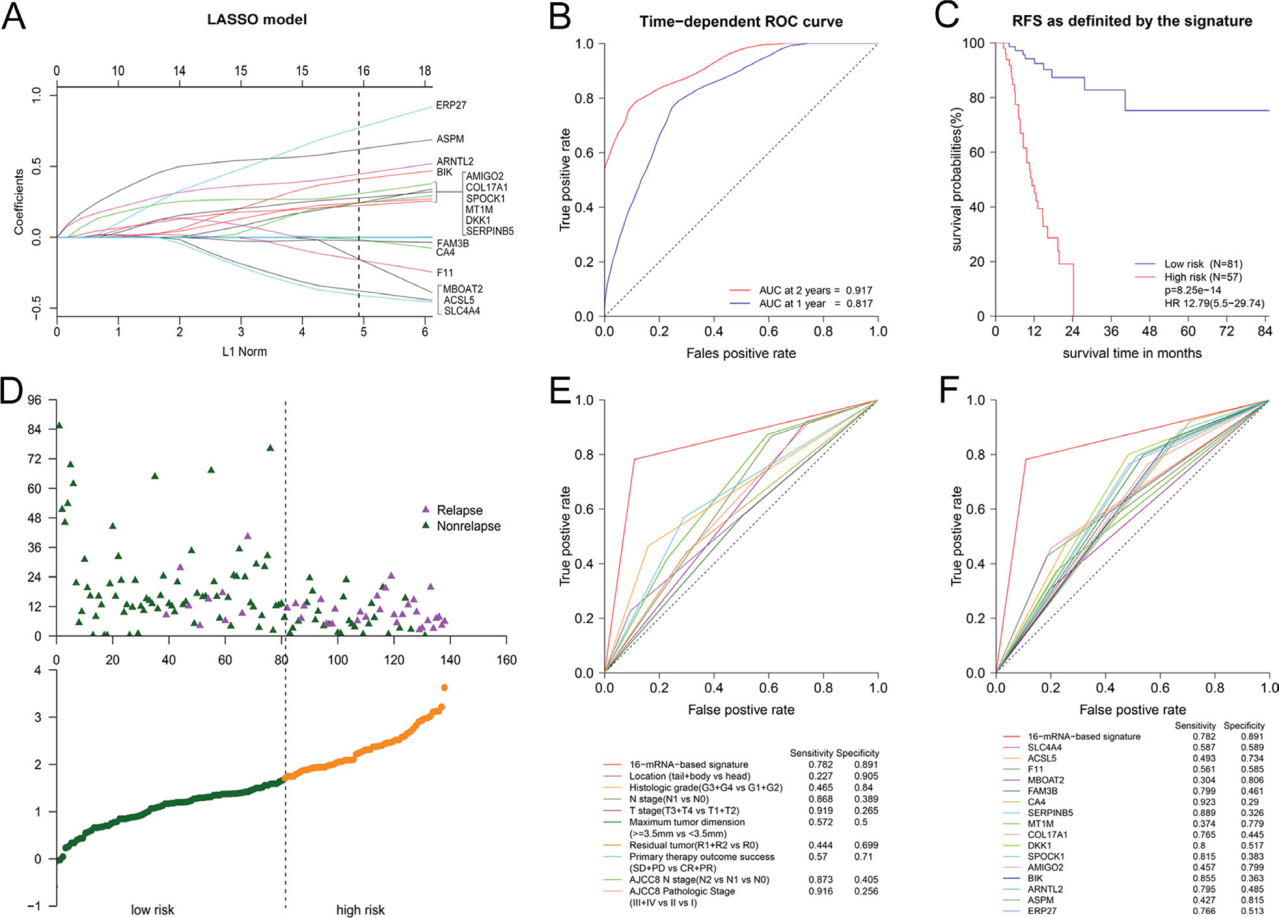
The 16-mRNA-based signature for predicting RFS (**A**) Visualization of the coefficient profiles of fitting LASSO COX model. Each curve represents a variable. It shows the path of its coefficient against the L1-norm of the whole coefficient vector at as λ varies. A vertical line is drawn at the value chosen by 10-fold cross-validation. The above axis: the number of nonzero coefficients at as λ varies. It represents the degrees of freedom for model. X-axis: L1 Norm, the summation of absolute nonzero coefficients at as λ varies. Y-axis: the values of nonzero coefficients at as λ varies. (**B**) Time-dependent ROC curve in 138 PDAC patients. (**C**) The Kaplan-Meier survival curve for patients in different risk groups. (**D**) Risk score analysis for patients. Top: the distribution of RFS status. Bottom: risk score of 16-mRNA-based signature. (**E**) Comparison of prognostic accuracy between the signature and clinical characteristics. (**F**) Comparison of prognostic accuracy between the signature and single mRNAs.

The function *glmnet* returned a sequence of models for us. We preferred the most widely used 10-fold cross-validation method to select the best one of them. As shown in Supplementary Figure 1, We plotted the partial likelihood deviance versus log (λ), where λ was the tuning parameter. Herein, a value λ = 0.04513 with log (λ) = −3.098 was chosen by 10-fold cross-validation via minimum criteria. Cross-validation was run up to 100 times. The cross-validated errors were averaged and the lambda.min with minimum mean cross validation error was still equal to 0.04513. So we obtained 16 variables with nonzero coefficients at the value λ chosen by cross-validation. These mRNAs included ERP27, ASPM, ARNTL2, BIK, AMIGO2, SPOCK1, DKK1, COL17A1, MT1M, SERPINB5, CA4, FAM3B, MBOAT2, F11, ACSL5 and SLC4A4 (Figure 3A). On the basis of these mRNAs, we successfully built a 16-mRNA-based signature. The signature risk score of every patient was computed according to the summation of 16 mRNAs expression status multiplied coefficient: risk score of 16-mRNA-based signature = (0.77120*status of ERP27) + (0.62081*status of ASPM) + (0.44536*status of ARNTL2) + (0.40868*status of BIK) + (0.30749*status of AMIGO2) + (0.27687*status of SPOCK1) + (0.24414*status of DKK1) + (0.24231*status of COL17A1) + (0.24071*status of MT1M) + (0.22296*status of SERPINB5) + (−0.01564*status of CA4) + (−0.02489*status of FAM3B) + (−0.15373*status of MBOAT2) + (−0.16052*status of F11) + (−0.37751*status of ACSL5) + (−0.40839*status of SLC4A4). In this formula, Low expression status was equal to 0 and high expression status was equal to 1.

The time-dependent ROC curve between the signature and RFS showed AUC at 2 years was 0.917 while AUC at 1 year was 0.817 (Figure 3B). It demonstrated that risk score value of 1.709 was the optimal cutoff point for predicting relapse, with sensitivity of 0.782 and specificity of 0.891. According to the cutoff value, we classified patients into two different subgroups, of which low-risk group had lower risk score and high-risk group had higher risk score. Figure 3C showed Kaplan-Meier curve in which high-risk group had poor RFS. 1 year RFS was 45% (95% CI: 31.6%−63.9%) for high-risk group and 92.5% (95% CI: 86.3%−99.1%) for low-risk group. Meanwhile, 2 year RFS was 0% for high-risk group in contrast to 87.4% (95% CI: 78.6%−97.1%) for low-risk group. The result of risk score analysis was displayed in Figure 3D. Besides, we noted that Youden’s index (= sensitivity + specificity−1) of 16-mRNA-based signature was higher than any clinical factor or single mRNAs when predict time was equal to 24 months (Figure 3E–3F). Furthermore, we analyzed the association of the 16mRNAs and the signature risk score with all clinical characteristics (Supplementary Figure 2). We found that the distribution of tobacco smoking history, histologic grade, T stage and residual tumor status differed between high-risk and low-risk group.

### Results of COX univariable analysis, multivariable analysis and stratified analysis

To verify that 16-mRNA-based signature had an excellent prognostic value, COX univariable analysis of 20 clinical factors and the signature with RFS were conducted. It demonstrated that 16-mRNA-based signature, histologic grade, N stage, T stage, primary therapy outcome success and AJCC8 N stage were significantly related to RFS (*p* < 0.05) (Table 2). We recognized that the hazard ratio for cancer relapse was 12.79 (95% CI: 5.5–29.74) when high-risk group compared to low-risk group (*p* < 0.0001). All *p* values were all calculated with Log-rank test here.

**Table 2:** Result of COX univariable analysis and multivariable analysis of RFS

Characteristics	Univariable HR (95% CI)	*P* value	Multivariable HR (95% CI)	*P* value
Gender (Female vs. male)	1.2 (0.64–2.26)	0.5615		
Age (≥65 years vs. <65)	1.01 (0.54–1.92)	0.9652		
History of diabetes (YES vs. No)	0.79 (0.36–1.75)	0.5578		
History of chronic pancreatitis (YES vs. No)	0.72 (0.22–2.39)	0.5943		
Family history of cancer (YES vs. No)	0.73 (0.34–1.56)	0.4186		
Tobacco smoking history (YES vs. No)	1.51 (0.73–3.09)	0.2625		
Alcohol history documented (YES vs. No)	1.39 (0.7–2.79)	0.3469		
Location (tail + body vs. head)	0.47 (0.18–1.23)	0.1161	0.35 (0.1–1.23)	0.1021
Histologic grade (G3 + G4 vs. G1 + G2)	2.54 (1.34–4.84)	**0.0031**	2.09 (0.87–4.98)	0.0973
Pathological stage (III + IV vs. II vs I)	1.55 (0.73–3.31)	0.257		
N stage (N1 vs. N0)	3.14 (1.3–7.56)	**0.0073**		
T stage (T3 + T4 vs. T1 + T2)	2.77 (0.96–7.94)	**0.0497**		
Maximum tumor dimension (≥3.5 mm vs. <3.5 mm)	1.66 (0.86–3.2)	0.1304	1.57 (0.71–3.51)	0.2662
Residual tumor (R1 + R2 vs. R0)	1.56 (0.77–3.14)	0.2095		
Radiation therapy (YES vs. No)	1.2 (0.61–2.35)	0.5895		
Targeted molecular therapy (YES vs. No)	1.14 (0.54–2.41)	0.7388		
Primary therapy outcome success (SD + PD vs. CR + PR)	2.42 (1.24–4.7)	**0.0074**	1.31 (0.61–2.83)	0.4885
AJCC8th T stage (T3 + T4 vs. T1 + T2)	1.37 (0.73–2.58)	0.3273		
AJCC8th N stage (N2 vs. N1 vs. N0)	1.59 (1.06–2.39)	**0.0236**	1.87 (0.74–4.73)	0.1828
AJCC8th Pathological Stage (III + IV vs. II vs I)	1.49 (0.95–2.32)	0.0784	0.92 (0.34–2.49)	0.8699
16-mRNA-based signature (High vs. Low)	12.79 (5.5–29.74)	**<0.0001**	7.74 (3.25–18.45)	**<0.0001**

AJCC: American Joint Committee on Cancer; CI: Confidence interval; CR: Complete remission /response; HR: Hazard ratio; PD: Progressive disease; PR: Partial remission/response; SD: Stable disease.

In the light of AJCC 8th version staging system’s more repeatable estimation and finer stratification, we performed multivariable analysis adjusted by clinical factors with *p* < 0.15 except AJCC 7th N stage and AJCC 7th T stage (Table 2). The adjusted hazard ratio was 7.74 (95% CI: 3.25–18.45, *p* < 0.0001), which indicated that 16-mRNA-based signature was still an independent prognostic factor for PDAC patients.

**Figure 4:**
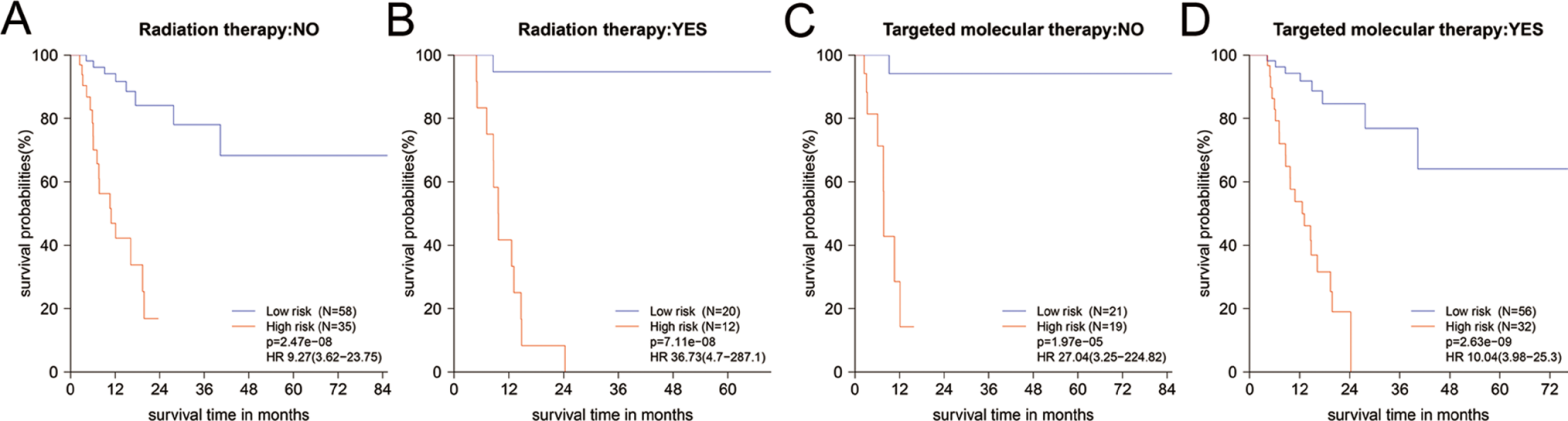
**(A–D)** The Kaplan-Meier survival curves for PDAC patients in different subgroups stratified by radiation and targeted molecular therapy. Information of partial patients on radiation or TMT was incomplete and deleted. TMT: targeted molecular therapy.

After stratified by clinical factors, 16-mRNA-based signature remained powerful for predicting RFS (Supplementary Table 6). In patients with the receipt of different treatment modalities, the results returned that high-risk group would have worse RFS in those whether received radiation or TMT (Figure 4, Supplementary Table 6). This was consistent with the result of Figure 3C. From Table 2, we noted that adjuvant radiation or TMT did not enhance survival in all patients (HR = 1.2, 95% CI: 0.61–2.35; HR = 1.14, 95% CI: 0.54–2.41, respectively). So we investigated the impact of radiation or TMT on RFS when these patients were stratified by 16-mRNA-based signature. However, we found no difference between radiation and non-radiation in whether high-risk group or low-risk group (Supplementary Figure 3A–3B). For low-risk group, it might be beneficial to treat with radiation, but might be bad to treat with TMT (Supplementary Figure 3A, 3C). They should receive postoperative radiation rather than TMT. High-risk group might potentially benefit from TMT, although the *p* value did not reach statistical significance (HR = 0.48, 95% CI: 0.20–1.14, *p* = 0.09, Supplementary Figure 3D).

## DISCUSSION

Improving survival outcomes in PDAC patients is an arduous and urgent task, which requires the development of several aspects, such as early detection and precision diagnostic of tumorigenesis and metastasis, more advances in risk stratification with the help of biomarkers and imaging techniques, and multiple effective therapies available for individualized treatment.

In the present study, we established a novel mRNA signature as a prognostic biomarker for RFS. To obtain these 154 DEGs, we performed overlapping analysis with a strategical and stepwise method. The time-dependent ROC curve and COX univariable analysis initially evaluated the prognostic value of these DEGs. Sixty-five mRNAs with AUC ≥ 0.55 and *p* < 0.25 were utilized to construct LASSO COX regression model. Ten-fold cross-validation helped us to choose the best one with the minimum mean cross validation error from a sequence of models. The 16-mRNA-based signature risk score has been proven to be an independent and powerful prognostic factor for PDAC patients. Moreover, our risk score might be able to predict which patients benefit from TMT or radiation. Low-risk group might be suitable for treatment with radiation and high-risk group might potentially benefit from TMT.

Many clinical factors decreasing RFS after operation for PDAC were identified, such as elevated serum CA19-9 [9, 10], perioperative blood transfusion [11, 12], higher histologic grade [9, 13], stage IV [11], lymph node metastasis [13–15] and lower lymphocyte to monocyte ratio [16]. However, unfavorable prognosis has not yet been improved. The present study showed that factors including histologic grade, AJCC7th N stage, AJCC7th T stage, primary therapy outcome success, AJCC8th N stage were associated with RFS. In term of PDAC staging, it is difficult to use imaging to distinguish extrapancreatic tissue from pancreas because of the proliferative reaction to tumor. To improve the repeatability and reproducibility of stage, AJCC staging system (8th edition) has canceled assessment of extrapancreatic invasion by using imaging as a criterion. Because 64 stage T3 tumors were down-staged to T2 (*n* = 63) and T1 (*n* = 1), the association between AJCC 8th T stage and RFS was not significant (*p* > 0.05). Besides, pathologic stage was not a prognostic factor for RFS (*p* > 0.05) in PDAC. This might be explained by that the distribution of subgroups was disproportionate and the sample size was not large enough. Nearly all patients had III stage tumors in stage III + IV group (94%), and stage IIb patients accounted for 82% in stage II group. The malignant degree of tumor biology and survival between stage IIb and III patients were close. On the other hand, a non-significant interaction between pathologic stage and RFS was influenced by other factors possibly.

The prognostic potential of carbohydrate antigens has been well documented in recent years. Preoperative serum CA19-9 levels are the most widely used and the only biomarker approved by the U.S. Food and Drug Administration. Although it can serve as a diagnostic and prognostic biomarker, but its practical value is limited [9, 17–20]. Kohei *et al.* declared that preoperative serum CA19-9 level (≥529/<529 U/mL) was an independent indicator for recurrence within 1 year after pancreatectomy. But it featured a poor prognostic value with sensitivity of 86.2% and specificity of 50% [9]. In contrast to CA19-9, the sensitivity and specificity of signature were 78.2% and 89.1% when predict time was two years. AUC at 2 years/1 year was 0.917/0.817. To the best of our knowledge, there is little in the literature about the predictive value of other serum carbohydrate antigens for RFS. Since other serum carbohydrate antigens are inferior to CA19-9 in the early diagnosis of PDAC, Yu *et al.* confirmed that preoperative serum CA19-9 level (≥37/<37 U/mL) had better predictive performance for OS and RFS than CA125 (≥18.6/<18.6 U/mL) level in PDAC patients [19]. But CA125 is superior to CA19-9 in hyperbilirubinemia patients with resectable PDAC. From the time-dependent ROC curves, we observed that AUC at 2 years/1 year for CA19-9 was approximately 0.68/0.65, which was less than ones of the 16-mRNA-based signature. On the other hand, postoperative and post-adjuvant chemotherapy serum CA19-9 levels (>37/≤37 U/mL) was all identified as an independent predictor for PDAC survival (HR = 2.697 and 2.72, respectively), regardless of preoperative serum CA19-9 level [20, 21]. Moreover, elevated postoperative serum CEA (>5.2/≤5.2 ng/mL) and CA125 (>35/≤35 U/mL) level further decreased RFS [20]. It provides us a train of thought that we can monitor and combine postoperative serum CA19-9, CEA with CA125 levels to ameliorate risk stratification in pancreatic cancer after resection. Additionly, metabolic tumor burden measured by 18F-FDG PET/CT is an effective prognostic factor for RFS, and superior to preoperative serum CA19-9, but PET/CT examination is rather expensive [10]. Elevated ERCC1 (HR = 2.1, 95% CI: 1.1–3.9) [22], DKK1 [23], CXCL12 [24], long non-coding RNA MALAT-1 (OR = 3.65, 95% CI: 1.86–7.18) [25] in tissue sample, and detection of circulating tumor cells (pooled HR: 2.36, 95% CI 1.41–3.96) [26] in peripheral blood were risky factors for patients survival. However, the 16-mRNA-based signature was significantly linked to RFS (HR = 7.74, 95% CI: 3.25–18.45).

Recent studies have proved that all the 16 mRNAs were differentially expressed between PDAC and normal pancreas tissue. Among these 16 mRNAs, 5 genes including ERP27, MT1M, CA4, F11 andFAM3B were down-regulated (Supplementary Table 5). ERP27 was shown to be down-regulated in acute pancreatitis in rats and selectively expressed in pancreatic exocrine glandular cells [27, 28]. Tumor suppressor MT1M promoter methylation was utilized to detect hepatocellular carcinoma (HCC) non-invasively in blood [29]. Recent study firstly stated that CA4 was a tumor suppressor in colorectal cancer (CRC) by inhibiting the Wnt signaling pathway. CA4 promoter methylation was an independent biomarker for the recurrence of CRC [30]. Moreover, renal cell carcinoma patients after nephrectomy with lower CA4 had an adverse survival [31]. The expression level of F11 (coagulation factor XI) in normal pancreas is comparable with that in liver [32]. FAM3B also called pancreatic-derived factor (PANDER) was secreted by pancreatic beta-cells and maintained glucose and lipid metabolism [33]. Another 11 genes were up-regulated. DKK1 was a WNT signaling pathway inhibitor whose promising diagnostic and prognostic value raised a continous concern in several tumors. It has been investigated that its high serological level was in connection with poor survival in CRC, laryngeal squamous cell carcinoma, HCC, lung cancer, esophageal carcinomas and PDAC [34–39]. It was also considered as a diagnostic biomarker in breast cancer and above mentioned cancers [34–40]. ASPM was connected with pancreatic epithelial tubulogenesis and over-expressed ASPM promoted aggressiveness of PDAC by regulating Wnt-β-catenin signaling pathway [41]. ARNTL2 was up-regulated and had the potential to be the marker for tumor aggressiveness in CRC [42]. BIK, one of Bcl-2 homology domain 3-only proteins was identified to induce apoptosis and predict breast cancer outcomes independently [43]. The pooled BH3-only proteins served as a novel prognostic biomarker in glioblastoma multiforme [44]. Up-regulation of AMIGO2 in fibrosarcoma cells promoted liver metastasis through the development of liver endothelial cell adhesion [45]. SPOCK1 acted as a potential prognostic factor for pregression and took part in tumor proliferation and metastasis through the ERK and AKT signaling pathways [46]. The hyper-methylation of COL17A1 promoter increased ductal breast cancer metastasis. But COL17A1 was over-expressed and its promoter was hypo-methylated in cervical cancer and other epithelial cancers [47]. SERPINB5 (also called maspin) served as an independent risky prognosticator for OS in PDAC patients after operation [48]. Previous studies have announced that over-expression of MBOAT2 was negatively related to PDAC patient survival [49]. ACSL5 involved in enterocytic differentiation and maturation has already been a predictive prognostic factor for early tumor recurrence in CRC patients [50]. SLC4A4 mRNA involved in Cl(–) and HCO(3)(–) efflux was selectively expressed in pancreatic ductal cells higher than in islet cells [28, 51].

Using LASSO model, Zhang *et al.* built a six-miRNA-based signature for identifying patients with stage II colon cancer who were suitable candidates for chemotherapy [52]. Ten-fold cross-validation was widely used to estimate how accurately a predictive model will perform in practice. The original samples were randomly classified into 10 equal sized subsamples, 9 subsamples were retained as training data while the remaining subsample was used as validation data. In this study, the 16-mRNA-based signature was at a minimum lambda with a minimum mean cross validation error. Transcript abundances of samples are got from RNA-seq rather than qRT-PCR. That is a limit of the study. But RNA-seq does not need to depend on reference gene for normalizing genes and is considered to be likely superior to qRT-PCR in the future [53]. GAPDH and β-actin are usually selected as reference genes because of their constant and abundant expression level. However, the difference of GAPDH expression levels was observed between TCGA recurrent and non-recurrent group, whilst the expression level of β-actin did not vary significantly (Supplementary Figure 4). We can analogize that GAPDH expression level may have a difference between two groups in qRT-PCR even if all experimental processes are perfect. So the error will be also fatal when we perform qRT-PCR and select incorrect reference gene. qRT-PCR is not unchallengeable. The reasons of our study only chose the mRNAs were that many studies showed a wide difference in differentially expressed miRNAs (DEmiRs). The relation of long non-coding RNAs to PDAC survival has not been well documented. The extraction protocols and detection methods of mRNAs, miRNAs and long non-coding RNAs were also different.

In summary, the novel mRNA signature can be a prognostic biomarker and has an excellent accuracy of predicting cancer recurrence for postoperative patients.

## MATERIALS AND METHODS

### PDAC datasets preparation

Gene expression profiles of PDACs were downloaded from public GEO (https://www.ncbi.nlm.nih.gov/geo/) and TCGA (http://cancergenome.nih.gov/). In the GEO Repository Browser, we retrieved 488 series with respective GSE identification numbers and a title containing the word “pancreatic”. We exported all searching results into a Microsoft Excel and picked out 297 datasets about homo sapiens species. Their summaries were reviewed carefully and 17 GEO datasets about human gene expression profiles of TvsP or TvsN were eligible. Exclusion criteria were unanalyzable datasets; failure to meet quality control standards: actin3/actin5 <3 and gapdh3/gapdh5 <1 assessed by the function *qc* in R package simpleaffy; the small number of of DEGs which was under 100; and incomplete annotated genes which accounted for less than 90% of genes within the total transcriptomes (*n* < 18000). Three datasets generated from non-mainstream platforms were unanalyzable and excluded. Four datasets have also been excluded due to other mentioned reasons (Supplementary Figure 5). In addition, TCGA database provided gene expression profile (level 3 data, log2(RSEM+1) transformed) from RNA-seq and corresponding clinical information in 138 PDAC patients with RFS status.

### Gene expression profiles processing

The raw cel files on affymetrix platform and the raw text file formats on agilent platform were gained. We used the function *ReadAffy* to read affymetrix microarray intensity data, and we conducted background correction, normalization and expression calculation with the function *Robust Multi-Array Average (RMA)* in the R package affy. Agilentmicroarray intensity data was read by using the function *read.maimages* in R package Linear Models for Microarray Data (LIMMA), and background correction, normalization and expression calculation were also completed by the package LIMMA. Boxplots were produced for observing whether scales of expression levels of microarrays were approximately equal. If multiple probes were annotated with the same gene, their mean value was computed to represent expression level of this common gene. The function *Fit linear model (lmFit)* in LIMMA was used to identify DEGs. Genes with adjusted *p* value (Adj.P.Val, also called false discovery rate, FDR) < 0.05 and absolute fold change (FC) > 2 were considered as DEGs. R packages ggplot2 were applied to draw Vocano plots for visualizing the results. Besides, overlapping genes were got from Venn diagram by means of the R package VennDiagram.

### GO enrichment analysis and KEGG pathway analysis

We utilized 154 highly up-regulated or down-regulated DEGs to perform GO enrichment analysis and KEGG pathway analysis in the R package clusterProfiler. Module categories with adjusted *p* value <0.05 were identified to be enriched significantly.

### LASSO regression model building

As we all know, Cox proportional hazards regression model is the most popular method of analyzing survival data. LASSO is a popular regression method and suitable for analyzing gene expression profile because microarray data has higher dimensionality, smaller sample size and strongly relevant variables [54]. Previous studies have applied it to Cox proportional hazards regression model broadly [52, 55]. We constructed LASSO COX regression model by utilizing several DEGs which expressed abnormally in PDAC and were related to prognosis. The function *glmnet* in package glmnet returned a sequence of lambdas (λs) and models for us. The value of the tuning parameter λ was negatively related to the complexity of the model and the value of deviance. As shown in Figure 3A, When the value of the invisible λ increased from left to right, the number of nonzero coefficients increased accordingly, and L1 Norm, the summation of absolute nonzero coefficients would become bigger. By using the function *cv.glmnet* in package glmnet, 10-fold cross-validation was conducted for us to choose the best model. Even though it was so strict, the results of *cv.glmnet* were slightly variable. We run the function *cv.glmnet* 100 times follow the glmnet reference manual’s advice and averaged cross-validation error curves. The lambda.min with minimum mean cross validation error was chosen by 10-fold cross-validation via minimum criteria. Then we run the function *glmnet* or *cv.glmnet* once more with the lambda.min and extracted variables with nonzero coefficients and their corresponding coefficients. R codes and the input file for LASSO COX regression model were offered in Supplementary Tables 7–8.

### Statistical analysis and graphics

All statistical analysis and graphics were performed in R software (R version 3.3.2). Time-dependent ROC curves were designed to compute the optimal cutoff value, sensitivity and specificity by using the package survival ROC. Kaplan-Meier survival curves were drawn to analyze the relationship between variables and RFS in the survival package. We used Pearson chi-squared test, corrected chi-squared test or Fisher’s exact test to examine the association of clinical characteristics with mRNAs. COX univariable analysis, multivariable analysis and stratified analysis were also performed in the package survival.

## SUPPLEMENTARY MATERIALS FIGURES AND TABLES











## Abbreviations

Adj.P.Val: Adjusted *p* value
AUC: Area under the curve
BP: Biological processes
CA199: Carbohydrate antigen 199
CC: Cellular component
CI: Confidence interval
CR: Complete remission/response
DEGs: Differentially expressed genes/mRNAs
DEmiRs: Differentially expressed miRNAs
ECM: Extracellular matrix
FC: Fold change
FDR: False discovery rate
GEO: Gene Expression Omnibus databases
GO: Gene Ontology
HCC: Hepatocellular carcinoma
HR: Hazard ratio
KEGG: Kyoto Encyclopedia of Genes and Genomes
LASSO: least absolute shrinkage and selection operator
LIMMA: Linear Models for Microarray Data
lmFit: Fit linear model
MF: Molecular functions
mRNAs: Messeger RNAs
NNE: Nearest neighbor estimation
OS: Overall survival
PC: Pancreatic cancer
PD: Progressive disease
PDAC: Pancreatic ductal adenocarcinoma
PLN: Positive lymph nodes
PorR: Protective or Risky factor
PR: Partial remission/response
RFS: Relapse-free survival
RMA: Robust Multi-Array Average
ROC: Receiver operating characteristic
SD: Stable disease
TCGA: The Cancer Genome Atlas database
TMT: Targeted molecular therapy
TvsP: Human PDACs versus paracancerous tissues
TvsN: Human PDACs versus normal pancreas tissues
