# Supplementary material for: A novel messenger RNA signature as a prognostic biomarker for predicting relapse in pancreatic ductal adenocarcinoma
Source: Oncotarget. 2017 Dec 2;8(67):110849–60. doi: 10.18632/oncotarget.22861 (PMC5762289; doi:10.18632/oncotarget.22861)
Supplement: Supplementary file 1 [file oncotarget-08-110849-s001.pdf]

A novel messenger RNA signature as a prognostic biomarker for predicting relapse in pancreatic ductal adenocarcinoma

SUPPLEMENTARY MATERIALS

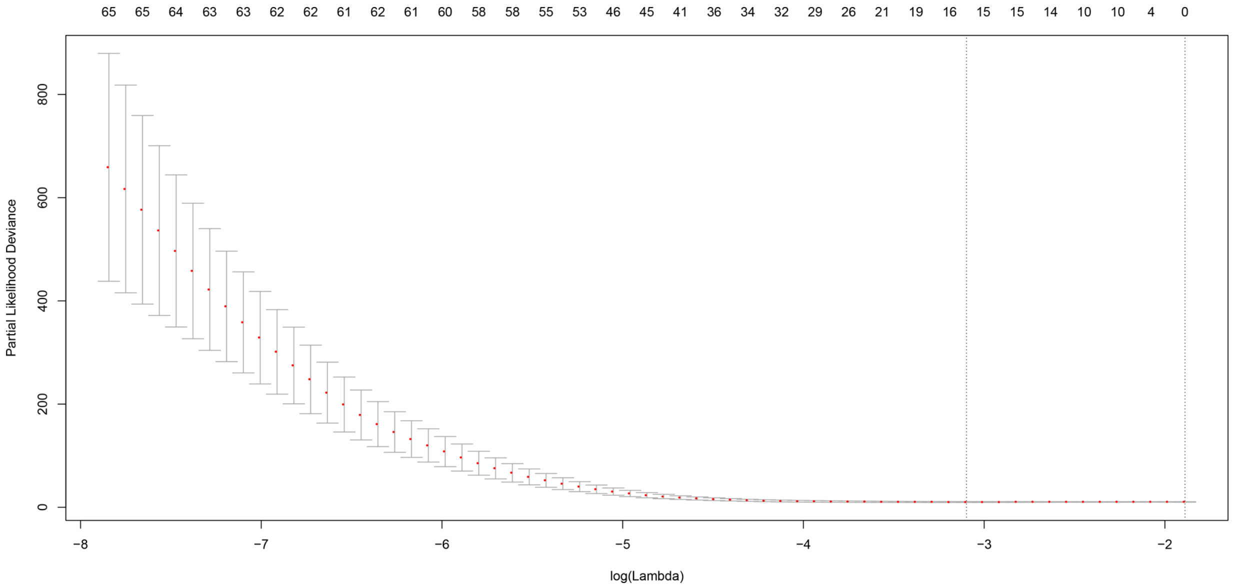

Supplementary Figure 1: 10-fold cross-validation for tuning parameter selection in the LASSO model. The solid vertical lines are partial likelihood deviance ± standard error (SE). The dotted vertical lines are drawn at the optimal values by minimum criteria (lambda.min, left vertical dotted line) and 1-SE criteria (lambda.1se, right vertical dotted line). We plot the partial likelihood deviance versus log (λ), where λ is the tuning parameter. Herein, a value λ = 0.04513 with log (λ) = –3.098 is chosen by 10-fold cross-validation via minimum criteria.

	SLC4A4	ACSL5	F11	MBOAT2	FAM3B	CA4	SERPINB5	MT1M	COL17A1	DKK1	SPOCK1	AMIGO2	BIK	ARNTL2	ASPM	ERP27	16-mRNA
Gender (Female/male)	0.251	0.686	0.62	0.193	0.334	0.284	0.879	0.253	0.876	0.404	0.134	0.516	0.647	0.498	0.18	0.542	0.239
Age (>=65 years vs <65)	0.13	0.536	0.859	0.77	0.373	0.647	0.276	0.24	0.148	0.039	0.24	0.28	0.489	0.121	0.549	0.952	0.343
History of diabetes (YES vs No)	0.506	0.732	0.861	0.943	0.855	0.665	0.993	0.456	0.494	0.364	0.686	0.494	0.478	0.874	0.635	0.599	0.871
History of chronic pancreatitis (YES vs No)	0.183	0	0.661	0.09	0.528	0.719	0.364	1	0.329	0.08	0.745	1	0.07	0.543	0.961	0.907	0.715
Family history of cancer (YES vs No)	0.314	0.716	0.379	0.987	0.118	0.824	0.792	0.731	0.895	0.846	0.603	0.025	0.937	0.31	0.773	0.487	0.907
Tobacco smoking history (YES vs No)	0.937	0.346	0.872	0.304	0.716	0.362	0.039	0.451	0.023	0.032	0.105	0.084	0.161	0.119	0.52	0.559	0.028
Alcohol history documented (YES vs No)	0.158	0.792	0.538	0.466	0.275	0.714	0.984	0.233	0.124	0.197	0.398	0.093	0.264	0.744	0.546	0.918	0.392
Location (tail+body vs head)	0.967	0.845	0.103	0.401	0.01	0.532	0.616	0.118	0.472	0.387	0.068	0.956	0.767	0.201	0.843	0.131	0.598
Histologic grade (G3+G4 vs G1+G2)	0.393	0.982	0.748	0.181	0.405	0.251	0.138	0.894	0.062	0.045	0.337	0.038	0.134	0.261	0.074	0.34	0.041
Pathologic stage (III+IV vs II vs I)	0.3	0.564	0.378	0.823	0.076	0.892	0.016	0.43	0.009	0.064	0.592	0.111	0.065	0.197	0.78	0.004	0.214
N stage (N1 vs N0)	0.815	0.476	0.931	0.538	0.925	0.489	0.061	0.659	0.023	0.322	0.441	0.001	0.246	0.131	0.261	0.05	0.284
T stage (T3+T4 vs T1+T2)	0.087	0.148	0.021	0.101	0.733	0.315	0.001	0.036	0.001	0.002	0.356	0.067	0.413	0.025	0.802	0.604	0.015
Maximum.tumor.dimension (>=3.5mm vs <3.5mm)	0.156	0.122	0.077	0.218	0.062	0.043	0.65	0.657	0.153	0.853	0.953	0.048	0.226	0.15	0.903	0.484	0.402
Residual tumor (R1+R2 vs R0)	0.572	0.195	0.665	0.017	0.97	0.211	0.013	0.628	0.007	0.149	0.917	0.084	0.023	0.59	0.216	0.184	0.029
Radiation therapy (YES vs No)	0.138	0.587	0.443	0.198	0.187	0.236	0.78	0.832	0.924	0.335	0.38	0.813	0.835	0.073	0.551	0.933	0.989
Targeted molecular therapy (YES vs No)	0.317	0.157	0.1	0.559	0.244	0.022	0.181	0.042	0.518	0.237	0.244	0.244	0.764	0.431	0.75	0.353	0.233
Primary therapy outcome success (SD+PD vs CR+PR)	0.354	0.552	0.588	0.824	0.796	0.106	0.132	0.245	0.567	0.372	0.252	0.665	0.506	0.33	0.303	0.967	0.275
AJCC8 T stage (T3+T4 vs T1+T2)	0.103	0.374	0.006	0.045	0.017	0.004	0.342	0.599	0.103	0.992	0.528	0.123	0.127	0.193	0.775	0.127	0.269
AJCC8 N stage (N2 vs N1 vs N0)	0.975	0.28	0.972	0.165	0.664	0.687	0.177	0.788	0.125	0.041	0.616	0.004	0.408	0.442	0.587	0.228	0.462
AJCC8 Pathologic Stage (III+IV vs II vs I)	0.922	0.435	0.35	0.196	0.422	0.877	0.125	0.814	0.011	0.049	0.831	0.005	0.057	0.682	0.743	0.346	0.288

Supplementary Figure 2: The association of 16 mRNAs and the signature risk score with clinical characteristics. Modules with p value <0.05 are marked with label colors. Pearson chi-squared test, corrected chi-squared test and Fisher's exact test are used.

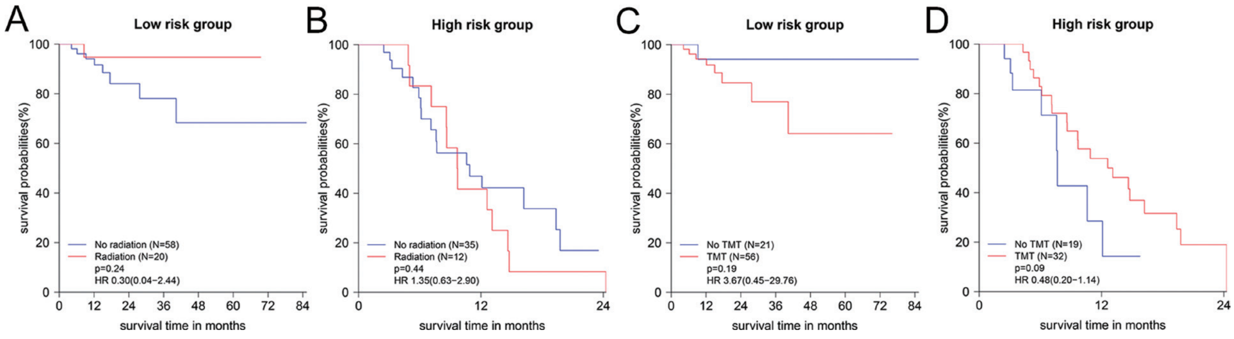

Supplementary Figure 3: The impact of radiation and TMT on RFS in different risk groups. (**A**–**D**) The Kaplan-Meier survival curves for PDAC patients in different subgroups stratified by 16-mRNA-based signature. TMT:targeted molecular therapy.

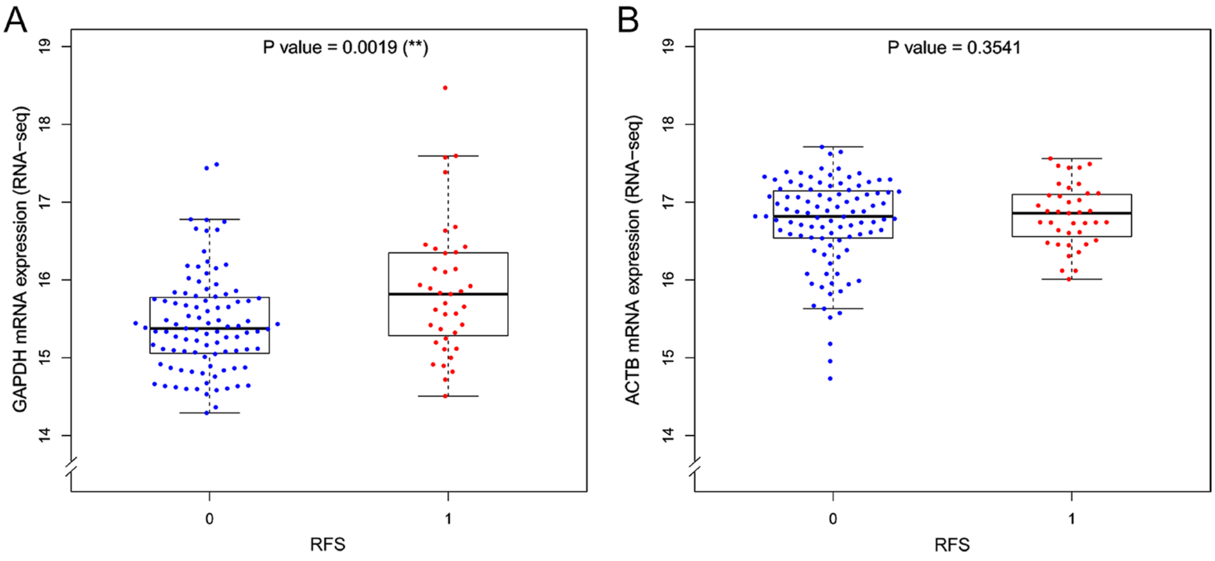

Supplementary Figure 4: GAPDH and ACTB expression levels in non-recurrent group and recurrent group.

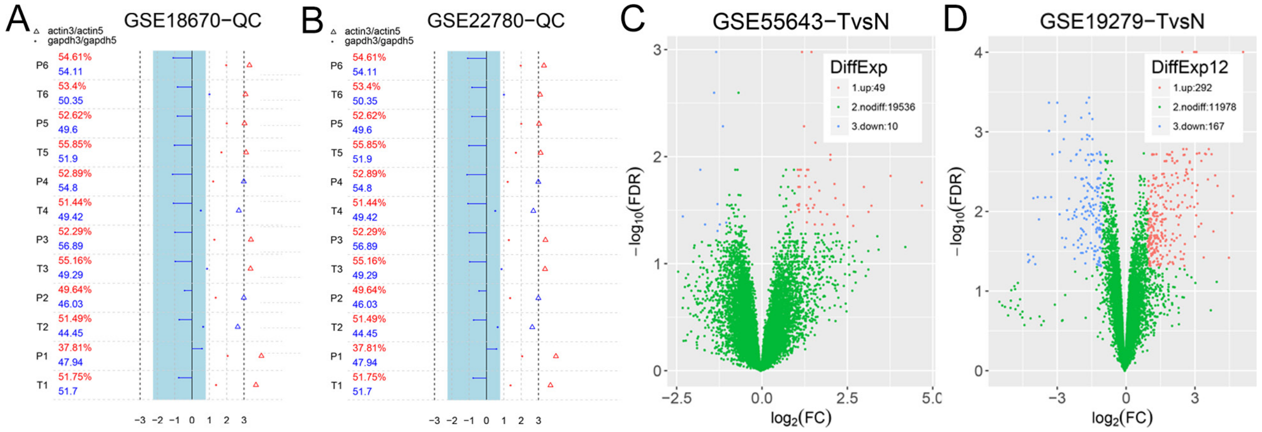

Supplementary Figure 5: Four datasets excluded from the study. (**A**–**B**) Quality control diagrams of two datasets. Two datasets (GSE22780 and GSE18670) do not meet quality control standards: actin3/actin5 < 3 and gapdh3/gapdh5 < 1. We use a function *qc* to detect RNA quality and confirm hybridisation efficiency in the R package simpleaffy. (**C**–**D**) Vocano plots of DEGs in another two datasets. GSE55643 only identifies relatively small number of DEGs. The number of DEGs in GSE55643 is fewer than 100. GSE19279 is annotated with 12437 incomplete genes. Exclusion criteria are unanalyzable datasets; failure to meet quality control standards: actin3/actin5 < 3 and gapdh3/gapdh5 < 1 assessed by the function *qc* in R package simpleaffy; the small number of of DEGs which is under 100; and incomplete annotated genes which accounts for less than 90% of genes within the total transcriptomes ($n < 18000$).

Supplementary Table 1: Clinical characteristics of 138 PDAC patients enrolled in the study

Characteristics	Number
All	138
Gender (male/female)	76/62
Age (<65/≥ 65 years)	66/72
History of diabetes(No/YES/NA)	78/32/28
History of chronic pancreatitis(No/YES/NA)	93/12/33
Family history of cancer(No/YES/NA)	39/51/48
Tobacco smoking history(No/YES/NA)	44/65/29
Alcohol history documented(No/YES/NA)	51/77/10
Location(head/body/tail/other)	106/9/12/11
Histologic grade(Gx/G1/G2/G3/G4)	1/26/71/38/2
Pathologic stage(I/Ia/Ib/IIa/IIb/III/IV/NA)	1/6/11/20/94/2/3/1
N stage(Nx/N0/N1/N1b/NA)	3/37/96/1/1
T stage(Tx/T1/T2/T3/T4)	1/8/17/110/2
Maximum tumor dimension(<35 mm/≥35 mm/NA)	62/70/6
Residual tumor(Rx/R0/R1/R2/NA)	4/83/45/1/5
Radiation therapy(No/YES/NA)	93/32/13
Targeted molecular therapy(No/YES/NA)	40/88/10
Primary therapy outcome success(CR/PR/SD/PD/NA)	58/8/6/40/26
AJCC8 T stage(T1/T2/T3/T4)	9/76/51/2
AJCC8 N stage(N0/N1/N2/NA)	38/55/42/3
AJCC8 Pathologic Stage(Ia/Ib/IIa/IIb/III/IV/NA)	7/18/12/53/44/3/1

AJCC: American Joint Committee on Cancer; CR: Complete remission /response; PD: Progressive disease; PR: Partial remission/response; SD: Stable disease.

Supplementary Table 2: T stage distribution of the AJCC 7th and 8th version staging system

8th edition	T1	T2	T3	T4	Total
**7th edition**					
Tx	0	1	0	0	1
T1	7	1	0	0	8
T2	1	11	5	0	17
T3	1	63	46	0	110
T4	0	0	0	2	2
total	9	76	51	2	138

Supplementary Table 3: N stage distribution of the AJCC 7th and 8th version staging system

8th edition	N0	N1	N2	NA	Total
**7th edition**					
Nx	0	0	0	3	3
N0	37	0	0	0	37
N1	1	55	41	0	97
NA	0	0	1	0	1
total	38	55	42	3	138

Supplementary Table 4: Pathologic Stage distribution of the AJCC 7th and 8th version staging system

8th edition	Ia	Ib	IIa	IIb	III	IV	NA	total
**7th edition**								
I	0	0	0	0	1	0	0	1
Ia	5	1	0	0	0	0	0	6
Ib	1	5	4	0	1	0	0	11
IIa	1	12	7	0	0	0	0	20
IIb	0	0	1	53	40	0	0	94
III	0	0	0	0	2	0	0	2
IV	0	0	0	0	0	3	0	3
NA	0	0	0	0	0	0	1	1
total	7	18	12	53	44	3	1	138

Supplementary Table 5: Results of time-dependent ROC curve analysis and COX univariable analysis. See Supplementary_Table_5

Supplementary Table 6: Result of stratified analysis of the 16-mRNA-based signature with RFS. See Supplementary_Table_6

Supplementary Table 7: R codes for LASSO COX regression model. See Supplementary_Table_7

Supplementary Table 8: The input file for running R codes for LASSO COX regression model. See Supplementary_Table_8
