# Supplementary material for: A novel messenger RNA signature as a prognostic biomarker for predicting relapse in pancreatic ductal adenocarcinoma
Source: Oncotarget. 2017 Dec 2;8(67):110849–60. doi: 10.18632/oncotarget.22861 (PMC5762289; doi:10.18632/oncotarget.22861)
Supplement: Supplementary file 4 [file oncotarget-08-110849-s004.docx]

**Table S7: R codes for LASSO COX regression model**

# read the file with csv format

> addicts <-read.csv('**Supplementary Table S8.csv**',row.names=1,header=T)

> dim(addicts) #nrow=138,ncol=87

> RT<-as.matrix(addicts[,23:87]) #65 mRNAs

> library(survival)

> y<-Surv(addicts[,2],addicts[,1]==1) #create a survival object

# run glmnet

> library("glmnet")

> fit<-glmnet(RT, y, family="cox")

> fit$lambda

> plot(fit)

# produce a PDF graphic:

> pdf(file="glmnet.pdf",width=16,height=8)

> plot(fit)

> dev.off()

# The function *glmnet* in package glmnet returned a sequence of lambdas (λs) and models for us. The value of the tuning parameter λ was negatively related to the complexity of the model and the value of deviance. When the value of the invisible λ increased from left to right, the number of nonzero coefficients increased accordingly, and L1 Norm, the summation of absolute nonzero coefficients would become bigger.


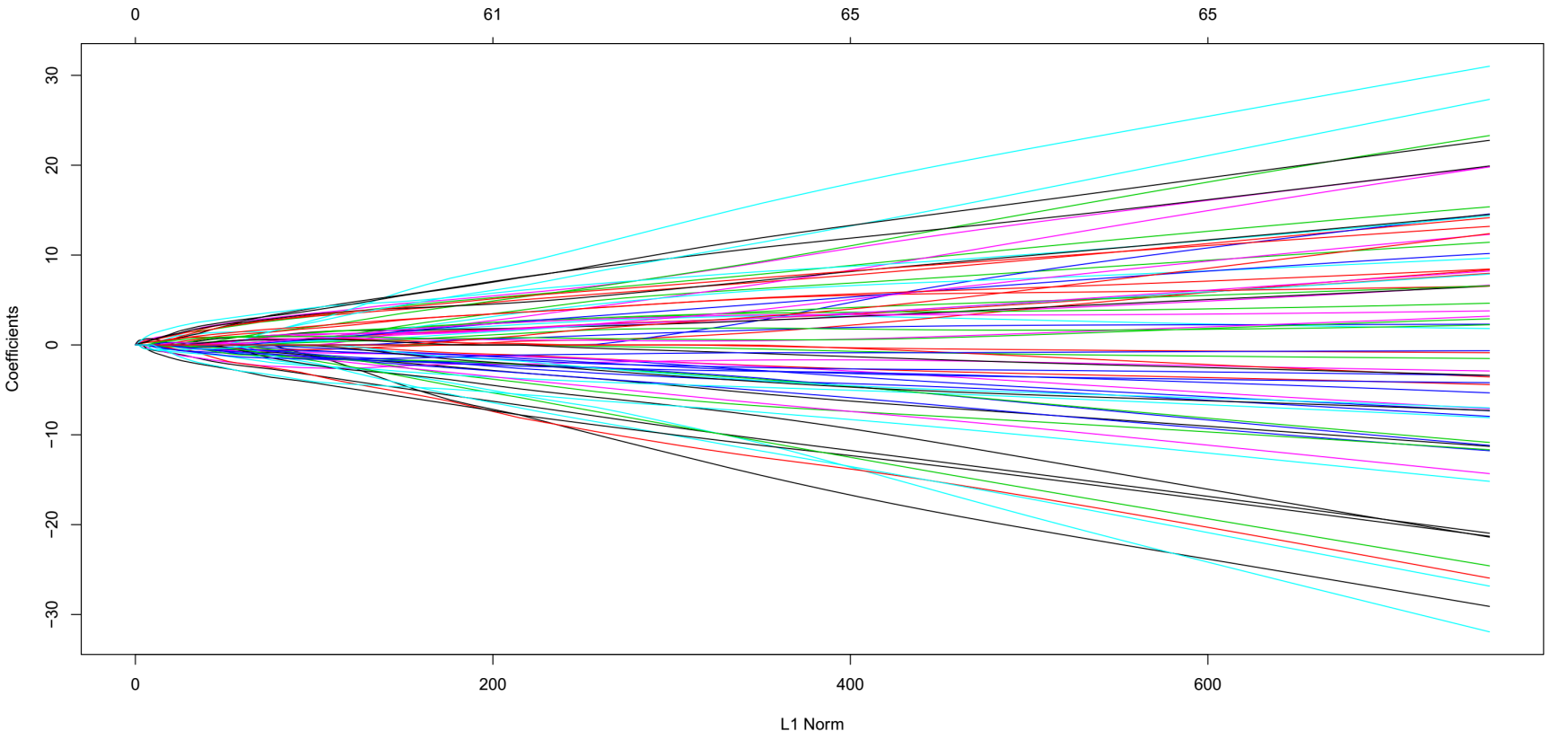


**Figure 1: Visualization of the coefficient profiles of fitting LASSO COX model.** Each curve represents a variable. It shows the path of its coefficient against the L1-norm of the whole coefficient vector at as λ varies. The above axis: the number of nonzero coefficients at as λ varies. It represents the degrees of freedom for model. X-axis: L1 Norm, the summation of absolute nonzero coefficients at as λ varies. Y-axis: the values of nonzero coefficients at as λ varies.

#The function *glmnet* returns a sequence of models for the users to choose from. In many cases, users may prefer the software to select one of them. Ten-fold cross-validation is the most widely used method for choosing the best model.

# run 10-fold cross-validation

> cvfit<-cv.glmnet(RT, y, family="cox", nfolds=10)

> cvfit$lambda

> cvfit$lambda.min #lambda.min is the value of λ that gives minimum mean cross-validated error..

> plot(cvfit)

# produce a PDF graphic:

> pdf(file="cvglmnet.pdf",width=16,height=8)

> plot(cvfit)

> dev.off()


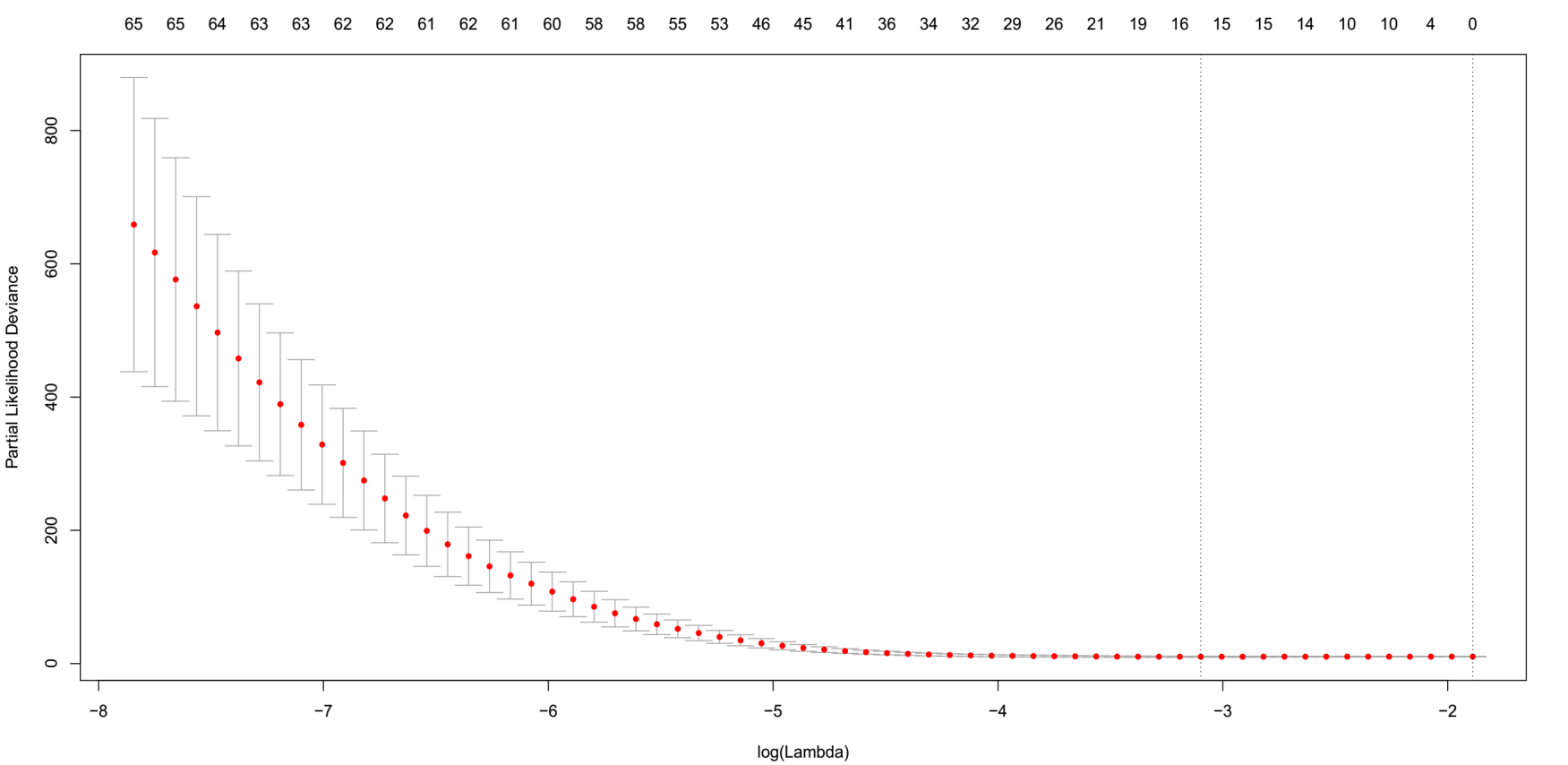


**Figure 2: 10-fold cross-validation for tuning parameter selection in the LASSO model.** The solid vertical lines are partial likelihood deviance± standard error (SE). The dotted vertical lines are drawn at the optimal values by minimum criteria (lambda.min, left vertical dotted line) and 1-SE criteria (lambda.1se, right vertical dotted line). We plotted the partial likelihood deviance versus log(λ), where λ is the tuning parameter. Herein, a value λ= 0.04513 with log(λ)= -3.098 was chosen by 10-fold cross-validation via minimum criteria.

# If you run 10-fold cross-validation once again, you would find that even though cross-validation was so strict, the results of cv.glmnet were slightly variable because λ.min (lambda.min) changed between several values. So we run *cv.glmnet* 100 times follow the glmnet reference manual’s advice.

# run the function *cv.glmnet* 100 times (taking a while)

> lambdas = NULL

> rm(errors)

> for (i in 1:100)

> {

> cvfit<-cv.glmnet(RT, Surv(addicts[,2],addicts[,1]==1), family="cox", nfolds=10)

> errors=data.frame(cvfit$lambda,cvfit$cvm) #cvm: the mean cross-validated error

> lambdas<-rbind(lambdas,errors)

> }

#take mean cvm for each lambda

> lambdas2<-aggregate(lambdas[, 2], list(lambdas[,1]), mean) #The mean cvm curves were averaged.

> colnames(lambdas2)<-c("lambda","cvm")

> write.csv(lambdas2,"138samples-65genes-lambda-cvm.csv",quote=F)

# select the best lambda

> best.index=which(lambdas2[2]==min(lambdas2[2]) )

> best.lambda=lambdas2[best.index,1]

> best.lambda #best.lambda is the value of λ that gives minimum mean mean cross-validated error.

# Cross-validation has been run 100 times. Each time, we produce one graphic. The mean cross-validated error curves are averaged and the best.lambda with minimum mean cross validation error is selected.

# obtain variables with nonzero coefficients and corresponding coefficients

> gt.coef<-coef(cvfit,s=best.lambda)

#The greater the value of given best.lambda is, the fewer coefficients the model contains. Herein, best.lambda with minimum averaged mean cross-validated error is selected after performing step 4.

> gt.coef[which(gt.coef != 0)] #variables with nonzero coefficients and matched coefficients

> sum(gt.coef[which(gt.coef != 0)]!=0) #the number of variables

> model<-matrix(data=NA, ncol=1, nrow=sum(gt.coef[which(gt.coef != 0)]!=0) )

> rownames(model)<-matrix(rownames(gt.coef)[which(gt.coef !=0)]) [,1]

> model[,1]<-matrix(as.numeric(gt.coef[which(gt.coef != 0)]))

> model<-as.matrix(model[order(model[,1]),])

> colnames(model)="coef"

> model

#Result:

[,1]

SLC4A4 -0.40838978

ACSL5 -0.37750577

F11 -0.16052206

MBOAT2 -0.15373253

FAM3B -0.02489483

CA4 -0.01564226

SERPINB5 0.22296552

MT1M 0.24071390

COL17A1 0.24231182

DKK1 0.24414427

SPOCK1 0.27686805

AMIGO2 0.30749143

BIK 0.40868182

ARNTL2 0.44536247

ASPM 0.62080675

ERP27 0.77120124

# compute risk scores

> classifier<-matrix(data=0,ncol=138,nrow=(dim(model)[1]+1))

> t<-t(RT)

> colnames(classifier)<-colnames(t)

> for(j in 1:138)

> for(i in 1:dim(model)[1])

> {classifier[i+1,j]=classifier[i,j]+model[i]*t[as.matrix(rownames(model))[i],j]}

> classifier[dim(model)[1]+1,]

# Risk score of 16-mRNA-based signature =(-0.40839* status of SLC4A4) +(-0.37751* status of ACSL5) +(-0.16052* status of F11) +(-0.15373* status of MBOAT2) +(-0.02489* status of FAM3B) +(-0.01564* status of CA4) +(0.22296* status of SERPINB5) +(0.24071* status of MT1M) +(0.24231* status of COL17A1) +(0.24414* status of DKK1) +(0.27687* status of SPOCK1) +(0.30749* status of AMIGO2) +(0.40868* status of BIK) +(0.44536* status of ARNTL2) +(0.62081* status of ASPM) +(0.77120* status of ERP27). In this formula, Low expression status equaled to 0 as well as high expression status equaled to 1.

# run glmnet again

> fit<-glmnet(RT, Surv(addicts[,2],addicts[,1]==1),family="cox")

> coef<-fit$beta

> coef.number<-fit$df

> L1Norm<-apply(abs(coef),2,sum)

> lambda<-rbind(L1Norm,coef.number)

> lambda

> rm(listcoef)

> for(i in 1:dim(lambda)[2])

> {if(lambda[1,i]>=1) break }

> listcoef<-lambda[2,i]

> for(i in 1:dim(lambda)[2])

> {if(lambda[1,i]>=2) break }

> listcoef<-c(listcoef,lambda[2,i])

> for(i in 1:dim(lambda)[2])

> {if(lambda[1,i]>=3) break }

> listcoef<-c(listcoef,lambda[2,i])

> for(i in 1:dim(lambda)[2])

> {if(lambda[1,i]>=4) break }

> listcoef<-c(listcoef,lambda[2,i])

> for(i in 1:dim(lambda)[2])

> {if(lambda[1,i]>=5) break }

> listcoef<-c(listcoef,lambda[2,i])

> for(i in 1:dim(lambda)[2])

> {if(lambda[1,i]>=6) break }

> listcoef<-c(listcoef,lambda[2,i])

> listcoef #10，14，15，15，16，18

> pdf(file="LASSO COX model fit.pdf")

> par(mar=c(10,4,10,4))

> plot(fit, bty="c",xaxs="i", lwd=0.5,font.axis=1,xlim=c(0,6.12) ,ylim=c(-0.5,1) )

> abline(v=sum(abs(gt.coef[which(gt.coef != 0)])) ,lwd=1.5, lty=2)

> dev.off()


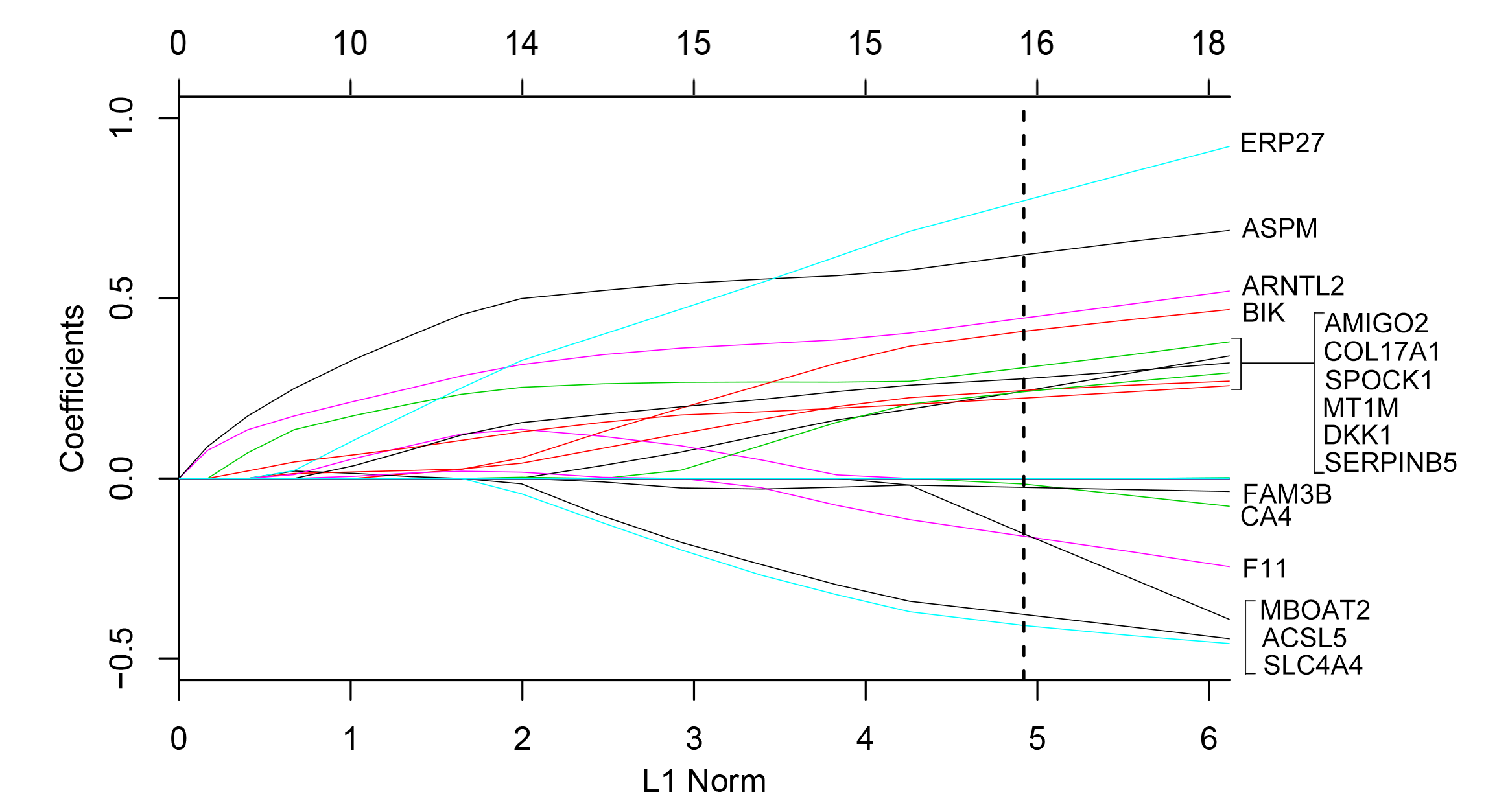


**Figure 3: Visualization of the coefficient profiles of fitting LASSO COX model.** Each curve represents a variable. It shows the path of its coefficient against the L1-norm of the whole coefficient vector at as λ varies. A vertical line is drawn at the value chosen by 10-fold cross-validation. The above axis: the number of nonzero coefficients at as λ varies. It represents the degrees of freedom for model. X-axis: L1 Norm, the summation of absolute nonzero coefficients at as λ varies. Y-axis: the values of nonzero coefficients at as λ varies.
